# Specialty Grand Challenge – Genetic Disorders

**DOI:** 10.3389/fped.2013.00036

**Published:** 2013-11-20

**Authors:** Jumana Y. Al-Aama

**Affiliations:** ^1^Department of Genetic Medicine, King Abdulaziz University, Jeddah, Saudi Arabia

**Keywords:** genetic disorders, medical disorders, genome, geneticist, adolescence

Medical Genetics is going through rapid growth with the help of evolving high throughput technologies and collaborative work across various scientific areas. Human genome sequencing opened the flood gate in identifying gene mutations for hundreds of genetic diseases and the genetic markers associated with complex disease risk factors. This decade can be considered as the golden age of medical genetics, which raises the biggest challenges for scientists from various fields, such as: Genetics, “Omics,” Bioinformatics, stem cell biology, and system biology, etc. to work together.

Human genome sequencing created massive amount of data and in turn led the way in identifying millions of common variants (SNPs) in the population (1). High throughput technologies using microarrays to screen millions of SNPs made it easy for scientists to carry out more than 1000 genome wide disease association (GWAS) studies and identify many disease associated markers (2). Whole exome sequencing (WES) and whole genome sequencing (WGS) are helping us to identify mutations in rare diseases (3). For example, whole exome analysis was exploited to its full, with many publications in high impact journals. Between 2010 and 2013, we have witnessed more rapid growth in identifying gene mutations in genetic diseases, especially rare diseases (4). These high throughput technologies brought together scientists across the globe to identify families with rare diseases and unravel previously unknown genetic secrets. The next stage of this era is moving toward application of these technologies in newborn screening and for diagnostics. This step faces multiple challenges in many areas before being introduced into the healthcare system (5, 6).

Newer technologies are being developed to make the analysis faster and more reliable. Scientists are caught in the web of catch up with the new technology to be the first ones to cash in on their beneficial effect. It is a never ending game that stimulates scientists and tries to address yet bigger challenges in this field. Success is slowly gaining momentum with this approach, but more collaboration will make the ultimate goal of patient care through science a reality.

High throughput data generation is accelerating so fast, that newer bioinformatics tools are needed to deal with large data. The informatics world is taking up the challenge. Hundreds of new bioinformatics tools are being developed to the ever changing high through put technologies. Close interaction of bioinformatics scientists with the bench scientists will provide the families and the clinicians with better options for patient care. Handling of whole exome and genome sequencing data requires a complex informatics pipeline and many groups are introducing tools which will tackle the large scale data generated by such high throughput technologies (7).

Every one of the gene mutation or SNP association solves one problem, but raises 10 questions in diagnosis, functional role, and treatment, etc. Diagnosis of the family at risk and development of accurate tests are the first step into the personalized patient care approach. It needs to be prioritized by the scientists. High throughput technologies in ’omics are being increasingly utilized to integrate genetics with ’omics data to understand the biological functions and pathways, etc. Functional understanding of the mutated genes or associated SNPs and their role in the human body is increasingly addressed by *in vivo* and *in vitro* methods (8). Geneticists are increasingly collaborating with scientists with different biological skills to unravel the functions of genes and how to modify (activate or inhibit) them to control or to treat patients.

Treating individuals by replacing defective genes is the ultimate challenge. Hopefully, this will be successfully addressed by the evolving technologies and newer discoveries in various scientific fields. Rapid publication of such important discoveries will disseminate the results faster to scientists around the world. High cost of health care across the globe, demands from families of affected patients and government organizations are pushing the pharmaceutical industry to make better medicines as well as personalized medicine for better patient care. Personalized Medicine and Pharmacogenetics are emerging fields. Success with personalized medicine is seen in many cancer treatments. Treatment of diseases is being explored using the functional effect of mutations, for example, successful CFTR activator treatment for a small group of cystic fibrosis patients with specific mutations (9). Functional understanding of causative genes will open the door for rare disease treatments (10).

Frontiers journal encourages scientists to submit their exciting work to address challenging questions raised in the field of Medical Genetics. Frontiers journals will join with the scientists to disseminate the new discoveries all over the world as early as possible for the benefit of patients.
